# The Envelope-Based Fusion Antigen GP120C14K Forming Hexamer-Like Structures Triggers T Cell and Neutralizing Antibody Responses Against HIV-1

**DOI:** 10.3389/fimmu.2019.02793

**Published:** 2019-12-04

**Authors:** Suresh C. Raman, Ernesto Mejías-Pérez, Carmen E. Gomez, Juan García-Arriaza, Beatriz Perdiguero, Aneesh Vijayan, Mar Pérez-Ruiz, Ana Cuervo, César Santiago, Carlos Oscar S. Sorzano, Cristina Sánchez-Corzo, Christiane Moog, Judith A. Burger, Anna Schorcht, Rogier W. Sanders, José L. Carrascosa, Mariano Esteban

**Affiliations:** ^1^Department of Molecular and Cellular Biology, Centro Nacional de Biotecnología, Consejo Superior de Investigaciones Científicas (CNB-CSIC), Madrid, Spain; ^2^Department of Structure of Macromolecules, Centro Nacional de Biotecnología, Consejo Superior de Investigaciones Científicas (CNB-CSIC), Madrid, Spain; ^3^X-ray Crystallization Unit, Centro Nacional de Biotecnología, Consejo Superior de Investigaciones Científicas (CNB-CSIC), Madrid, Spain; ^4^Biocomputing Unit, Centro Nacional de Biotecnología, Consejo Superior de Investigaciones Científicas (CNB-CSIC), Madrid, Spain; ^5^INSERM U1109, Fédération Hospitalo-Universitaire (FHU) OMICARE, Fédération de Médecine Translationnelle de Strasbourg (FMTS), Université de Strasbourg, Strasbourg, France; ^6^Department of Medical Microbiology, Amsterdam University Medical Centers, University of Amsterdam, Amsterdam, Netherlands; ^7^Department of Microbiology and Immunology, Weill Medical College of Cornell University, New York, NY, United States

**Keywords:** GP120C14K, VACV 14K protein, MVA-HIV vaccine, CD8 T cells, GC B cells, Tfh cells, SOSIPs, HIV-1 neutralizing antibodies

## Abstract

There is an urgent need for the development of potent vaccination regimens that are able to induce specific T and B cell responses against human immunodeficiency virus type 1 (HIV-1). Here, we describe the generation and characterization of a fusion antigen comprised of the HIV-1 envelope GP120 glycoprotein from clade C (GP120C) fused at its C-terminus, with the modified vaccinia virus (VACV) 14K protein (*A27L* gene) (termed GP120C14K). The design is directed toward improving the immunogenicity of the GP120C protein through its oligomerization facilitated by the fused VACV 14K protein that results in hexamer-like structures. Two different immunogens were generated: a recombinant GP120C14K fusion protein (purified from a stable CHO-K1 cell line) and a recombinant modified vaccinia virus Ankara (MVA) poxvirus vector expressing the GP120C14K fusion protein (termed MVA-GP120C14K). The GP120C14K fusion protein is recognized by broadly neutralizing antibodies (bNAbs) against HIV-1. In a murine model, a heterologous prime/boost immunization regimen with MVA-GP120C14K prime followed by adjuvanted GP120C14K protein boost generated stronger and polyfunctional HIV-1 Env-specific CD8 T cell responses when compared with the delivery of the monomeric GP120C form. Furthermore, the immunization protocol MVA-GP120C14K/GP120C14K elicited higher HIV-1 Env-specific T follicular helper cells, germinal center B cells and antibody responses than monomeric GP120. In addition, a similar MVA-GP120C14K prime/GP120C14K protein boost regimen performed in rabbits triggered high HIV-1-Env-specific IgG binding antibody titers that were capable of neutralizing HIV-1 pseudoviruses. The extent of HIV-1 neutralization was comparable to that elicited by the current standard GP140 SOSIP trimers from clades B and C when immunized as MVA-SOSIP prime/SOSIP protein boost regimen. Overall, the novel fusion antigen and the corresponding immunization scheme provided in this report can therefore be considered as potential vaccine strategies against HIV-1.

## Introduction

The development of an effective vaccine against human immunodeficiency virus type 1 (HIV-1) has been hampered by several inherent features of the virus-host interaction that makes it a difficult target. The scientific search for an ideal vaccine candidate antigen that elicits potent immunogenic response, both in terms of quantity and quality, against the virus is an active area of research. Thus, far, the efforts toward designing and developing such an antigen have largely been driven by the previous vaccination efforts, including the RV144 phase III clinical trial that, for the first time, provided a modest protection of 31.2% against the virus (1).

It is extensively clear that the conformational integrity of the GP120 protein plays a crucial role in influencing the nature of anti-HIV-1 responses. Therefore, there is a need for the use of modified HIV-1 Env antigens capable of displaying several desired immunological characteristics as vaccine candidates. Several attempts have been made to mimic the native conformation of the HIV-1 envelope through modifications of the protein structure. One such strategy has led to the development of native-like SOSIP trimers, where the trimeric portion of the envelope is maintained through modifications including the engineering of a disulphide bond between the GP120 and the GP41 residues in order to maintain the furin cleavage site (2). Several immunizations with the SOSIP trimer candidates in animal models have been conducted so far, indicating the ability of these constructs to raise broadly neutralizing antibodies (bNAbs) against the autologous tier-2 and heterologous tier-1 viruses (3). Although not sufficient to count as a vaccine success, the generation of these autologous tier-2 responses provides a good starting point for further exploration of the capacity of these SOSIP constructs in mounting a strong immune response capable of broadly neutralizing HIV-1.

With the aim to enhance the potency, breadth, and polyfunctionality of HIV-1 Env protein, in this investigation, we used a modified vaccinia virus (VACV) 14K protein (encoded by the *A27L* gene) as an oligomer-driven fusion agent for modifying the HIV-1 GP120 from clade C to form a novel antigen termed GP120C14K. The idea behind the implementation of the 14K oligomer fusion agent is to make use of the adjuvant-like effect that it confers to the vaccination regimen and to especially those involving poxvirus-based vectors. This has been demonstrated in the case of malaria, where fusion of the 14K molecule with the circumsporozoite (CS) *Plasmodium falciparum* antigen generated an oligomeric CS14K form that markedly improved the poxvirus-based vaccination protocol, including the inhibition of the liver-stage development of the malaria parasite leading to sterile protection in mouse models (4). Following similar approach, fusion of a modified version of the 14K molecule to the GP120 segment from clade B (isolate BX08) produced an oligomeric protein GP120-14K (5) that displayed in mice better antigenic characteristics than its GP120 monomeric counterpart. A prime with the DNA vector expressing the clade B GP120-14K fusion antigen followed by a boost using the HIV-1 vaccine candidate MVA-B (6) showed significant improvements in the HIV-1 specific CD4 and CD8 T cell responses compared to the use of a DNA priming agent expressing the monomeric GP120 antigen from the same clade B (7).

Encouraged by these improvements that the fusion with the 14K protein brought out over the HIV-1 antigen GP120, we decided to extend those findings and explore whether the clade C GP120C14K fusion antigen could be used to improve the immunogenicity of the GP120C molecule by oligomerization, providing an adjuvant-like effect capable of increasing the HIV-1-specific cellular and humoral immune responses. This has been accomplished through the generation of two forms of immunogens, one as a purified GP120C14K protein component produced in CHO cells and the other as a poxvirus-vector based on modified vaccinia virus Ankara (MVA) expressing GP120C14K. Here, we have characterized *in vitro* the fusion protein component and we established *in vivo* immunization protocols consisting of MVA-GP120C14K prime/GP120C14K protein boost that induced in mice high and broad T and B cell immune responses against HIV-1. The immune parameters induced, like activation of Env-specific CD8 T cells, T follicular helper (Tfh) cells, Germinal Center (GC) B cells and production of NAbs against HIV-1, might be relevant for protection against HIV-1. Moreover, immunization protocols involving MVA-GP120C14K based prime and GP120C14K protein boost induced in rabbits high levels of Env specific IgG antibodies and also NAbs against HIV-1 comparable to those induced by similar protocols involving the SOSIP proteins, known for their HIV-1 envelope native-like conformations.

## Materials and Methods

### Cells and Viruses

CHO-K1 cells used for protein production were grown in minimum essential medium (MEM) lacking glutamine in the presence of 25 μM of the negative selective agent L-methionine sulfoximine (MSX) (Sigma-Aldrich) and supplemented with 3% fetal calf serum (FCS). Established chick DF-1 cells (a spontaneously immortalized chicken embryo fibroblast (CEF) cell line; ATCC, Manassas, VA) and primary CEF cells were grown in Dulbecco's modified Eagle's medium (DMEM) supplemented with 10% heat-inactivated FCS. Cell cultures were maintained at 37°C (CEF and CHO-K1) or 39°C (DF-1) in a humidified incubator containing 5% CO_2_. MVA viruses (MVA-wt, MVA-GP120C, MVA-GP120C14K, MVA-AMC011, MVA-ZM197, and MVA-GPN) were generated as crude working stocks (P2) and further grown in primary CEF cells, purified by centrifugation through two 36% (w/v) sucrose cushions in 10 mM Tris-HCl pH 9 (8, 9), and titrated in DF-1 cells by immunostaining plaque assay, as previously described (6, 10, 11). The titer determinations of the different viruses were performed at least two times.

### Plasmid DNA Vectors

pBJ5-GS-GP120C and pBJ5-GS-GP120C14K vectors were generated by cloning the GP120C and GP120C14K (fusion of *GP120C* gene from clade CN54 at the C terminal with the modified VACV *A27L* gene lacking the first 28 amino acids) inserts, respectively, into the pBJ5-GS plasmid (kindly provided by Prof. José María Casasnovas, CNB-CSIC) which contains a glutamine synthetase minigene. These plasmids were used for the construction of stable CHO-K1 cell lines expressing the recombinant proteins GP120C or GP120C14K. pcDNA-GP120C and pcDNA-GP120C14K vectors are pcDNA3.0(+)-based plasmids with the GP120C or GP120C14K antigens inserted into the multiple cloning site (between *EcoRI* and *XbaI* restriction sites), used for vaccination and transient transfections. The pCyA20-GP120C14K transfer vector was generated by inserting the GP120C14K antigen in the pCyA20 plasmid transfer vector backbone (12), and was used for the insertion of the GP120C14K gene into the Thymidine Kinase (TK) locus of the MVA genome. It contains the GP120C14K fusion gene between the VACV TK-L and TK-R flanking regions along with the selectable marker genes for ampicillin and β-galactosidase (*LacZ* gene). The *LacZ* gene is inserted among two repetitions of the left TK-flanking region, allowing for its eventual deletion by homologous recombination after consecutive plaque purification. All plasmids were purified through Endofree Plasmid Megaprep Kit (Qiagen) following manufacturer's instructions.

### Generation of Stably Transfected CHO-K1 Cell Lines Expressing GP120C14K or GP120C

In order to generate CHO-K1 cells expressing GP120C14K or GP120C proteins in a stable fashion, the cells were transfected with pBJ5-GS-GP120C14K and pBJ5-GS-GP120C plasmids, respectively, using standard calcium phosphate transfection methods. Clones expressing GP120C14K or GP120C proteins were selected by supplementing the medium with MSX scaled-up. The resulting stably transfected CHO-K1 cell lines were grown in 1,450 cm^2^-roller flasks at 37°C while on a slow and constant rotation at about 0.5–1 rpm. The media containing the secreted protein was collected every 3–4 days depending on the nature and confluence of the cells.

### Protein Purification

Purification of HIV-1 GP120C14K and GP120C proteins was performed using lectin columns. Briefly, supernatants containing the protein of interest were centrifuged (4,000 rpm for 30 min at 4°C), supplemented with 0.05% sodium azide (Sigma-Aldrich) and frozen at −20°C until used. Prior to purification, the supernatants were thawed and filtered using 0.4-micron filters (Millipore). One liter of clarified supernatant was passed through Agarose bound *Galanthus nivalis* lectin columns (Vector Labs) at a rate of roughly 0.2 ml/min using gravity flow. The column was then washed with 15 ml of cold PBS and the elution was carried out with 25 ml of 0.5 M methyl-α-D-manno-pyrannoside (Sigma-Aldrich) at a rate of 0.2 ml/min. Positive fractions were collected, concentrated and their buffer was changed to 10 mM Tris pH 7.5, 150 mM NaCl using 100 kDa centrifugal concentrators (Millipore). Later, the proteins were separated through a Superdex-200 10/300 GL and Superdex-200 analytical Size Exclusion Chromatograms (SEC) according to manufacturer's instructions (GE Healthcare). The proteins were quantified using NanoDrop (Thermo Scientific, Wilmington, USA) and stored in aliquots at −20 or −80°C, depending on the period of storage. Aliquots for *in vivo* experiments were subjected to Lipopolysaccharide (LPS) determination using the Limulus Amebocyte Lysate (LAL) kit (QCL-1000, Lonza) according to manufacturer's instructions to ensure that the LPS content fulfills EU regulations (13). AMC011 SOSIP.v5.2 (from HIV-1 clade B) and ZM197 SOSIP.v5.2 (from HIV-1 clade C) proteins were generated and purified as previously described (14, 15).

### Native Gel Electrophoresis

Separation of larger molecular weight proteins under native conditions was carried out in standard 4–15% Mini-PROTEAN TGX gradient gels (Biorad). The samples were prepared using the Native Page sample buffer (Invitrogen) prior to separation. NativeMark Unstained Protein Standard (Thermo Fisher) was used for estimation of protein molecular weights. Staining was done using colloidal silver using the PlusOne Silver staining kit (GE Healthcare) according to manufacturer's instructions.

### Stability and Oligomerization State Analysis of Proteins

Protein stability measurements and oligomerization state analysis of GP120C14K were undertaken as part of an iNEXT structural audit at the protein facility of the Netherlands Cancer Institute (NKI), Amsterdam. Briefly, the protein samples were resuspended in various buffer conditions and the denaturation midpoint or Tm was measured using a Thermofluor assay based on previously described methods (16). The capacity to form aggregates was measured under the same buffer conditions using a Prometheus instrument (Nanotempertech) according to manufacturer's instructions. The oligomerization state of protein was analyzed using a size exclusion coupled multi angle light scattering (SEC-MALLS) assay according to previously described protocols (17).

### GraFix of GP120C14K for Negative-Staining Electron Microscopy (EM)

Stabilization of GP120C14K samples was carried out by Gradient Fixation (GraFix) method (18). During this process, the macromolecular complex was separated in a 10–40% (v/v) glycerol gradient in 50 mM HEPES pH 7.5, 150 mM NaCl, and 0.15% glutaraldehyde by ultracentrifugation for 16 h at 32,000 rpm using a SW55Ti rotor (Beckman). The glycerol gradient was prepared in a Gradient Master (Biocomp, Fredericton, NB, Canada) and after centrifugation the fractions were collected and the cross-linking reactions were stopped with 80 mM glycine.

### EM and Image Processing

Electron microscopy of negatively stained samples was performed as described previously (7). Briefly, GP120C14K molecules were applied onto carbon-coated copper grids and stained with 2% uranyl acetate. Micrographs were taken under loose dose conditions in a JEOL JEM1010 microscope operated at 80 kV, at 50,000× magnification and acquired in a 4K × 4K digital TemCam_F416 camera (TVIPS) with a pixel size of 2.53 A/pix. Particles were automatically selected using XMIPP3.1 (19–21) and homogeneous image populations were obtained after 2D classification with RELION (22, 23). A 25 Å resolution final model was obtained from 16,600 selected particles applying C3 symmetry using a RANSAC result as initial volume (24).

### Enzyme-Linked Immunosorbent Assay (ELISA)

In order to analyse the reactivity of the proteins (GP120C14K, GP120C, BG505-SOSIP) to standard bNAbs against HIV-1 envelope, 96-well Maxisorp plates (Nunc) were coated with 2 μg/ml of the purified protein in PBS 1X and incubated at 4°C overnight. The next day, the plates were blocked with 5% non-fat dry milk prepared in PBS 1X-0.01% Tween20 (PBS-T) (Sigma Aldrich) and washed three times with PBS-T. Serial dilutions of the corresponding bNAbs (PGT151, PGT121, 257-D IV, 10-1074) were prepared in PBS-T with 1% non-fat dry milk and added to the protein-coated plates. After 90 min of incubation at room temperature, the plates were washed three times with PBS-T and 1:1,000 dilution of secondary antibody (mouse α-human horseradish peroxidase; Sigma-Aldrich) prepared in PBS-T with 1% non-fat dry milk was added to the wells and incubated for 1 h at room temperature. After three final washes, the plates were developed by adding TMB substrate (3,3′,5,5′ Tetramethylbenzidine; Sigma-Aldrich), the reaction was stopped by adding H_2_SO_4_, and the absorbance at 450 nm was determined using Biochrom EZ Read 400 Microplate Reader (Biochrom Ltd., Cambridge, UK).

The anti-HIV-1 Env antibody levels present in the serum of immunized animals (mice or rabbits) were also determined by ELISA following similar methods. In this case, serial dilutions of the sera obtained from the animals were added to 96-well Maxisorp plates (Nunc) previously coated with 2 μg/ml of the Env protein of interest and incubated for 2 h. The endpoint dilution titer for each serum sample was calculated as the last dilution which gave an absorbance value >3 times the value of the absorbance given by the corresponding dilution of the naïve serum.

### Generation and *in vitro* Characterization of Recombinant Virus MVA-GP120C14K

In order to generate the recombinant viral vector MVA-GP120C14K, 3 × 10^6^ DF-1 cells were infected with 0.01 plaque forming unit (PFU)/cell of the wild type MVA virus (MVA-wt). After 1 h of virus adsorption, the inoculum was removed, the cells were washed with OPTIMEM media (Gibco) and then transfected with 8 μg of the pCyA20-GP120C14K plasmid transfer vector using lipofectamine reagent (Invitrogen) following manufacturer's recommendations. The DNA/liposome mixture was removed 5 h post-transfection, the cells were washed twice with OPTIMEM media (Gibco) and finally complete DMEM-2% FCS medium was added, and cells were incubated at 37°C with 5% CO_2_. The cells were harvested when extensive cytopathic effect was observed, centrifuged (1,800 rpm for 5 min), resuspended in 1 ml of DMEM and lysed by three freeze-thaw cycles. The extract thus obtained was sonicated before use (three cycles of 10 s sonication with a 10 s pause using the Misonix Incoporated S3000-010 sonicator). Then, DF-1 cells grown in 6 multi-well plates (Nunc) were infected for 1 h with serial dilutions of this cell extract and after the inoculum was removed, 3 ml of 1:1 agar (1.9%) and DMEM 2X-4% FCS was added and incubated at 37°C with 5% CO_2_. At 48 h post-infection, 0.03% of 5-bromo-4-chloro-3-indolyl β-D-galactopyranoside (X-Gal) in 1 ml of agar (1.9%) and DMEM 2X-4% FCS (1:1) was added (25, 26). Those plaques that developed blue color corresponding to the recombinant viruses were picked and resuspended in 0.5 ml DMEM and used as inoculum for further rounds of infection in DF-1 cells. This process of blue plaque selection was repeated three times, followed by three more rounds where non-colored plaques (corresponding to those recombinant viruses that have lost the selection marker gene) were picked. The correct insertion of the GP120C14K fusion antigen within the MVA genome was confirmed by PCR and DNA sequencing.

### MVA-GP120C14K Growth Kinetics and Stability

The replication capacity of MVA-GP120C14K recombinant virus was determined through a growth curve experiment performed in DF-1 cells. Cells were infected at 0.01 PFU/cell with viruses (MVA-GP120C14K, MVA-GP120C, or MVA-wt) for 1 h at 37°C, following which the inoculum was removed and DMEM-2% FCS was added. At various time points post-infection [0, 24, 48, 72, and 96-h post-infection (hpi)], the infected cells were harvested, lysed by three freeze-thaw cycles, sonicated and titrated in DF-1 cells. The stability of the MVA-GP120C14K recombinant virus was confirmed after nine successive passages in DF-1 cells at low virus multiplicity (0.01 PFU/cell) and analysis by Western blot of cell extracts derived from 32 individual virus plaques picked from virus at passage 9 and grown previously in DF-1 cells.

### Time-Course Expression of GP120C14K in MVA-GP120C14K-Infected Cells

Time-course expression of GP120C14K antigen by the MVA-GP120C14K virus was determined by Western blot. DF-1 cells were infected with MVA-GP120C14K virus at 5 PFU/cell and harvested at different times (6 and 24 hpi). Infected cells were collected, and the cells extracts, and supernatants were fractionated by 10% SDS-PAGE and then analyzed by Western-blot using rabbit anti-GP120 antibody (1:3,000; CNB) and C3 anti-14K antibody (1:3,000; CNB), and goat anti-rabbit-HRP (1:5,000; SIGMA) as secondary antibody. Detection was performed using an enhanced chemiluminescence system (ECL, GE Healthcare, Chicago, IL, USA).

### Generation of MVA Vectors Expressing GP140 AMC011 and ZM197 SOSIP Proteins

For the generation of MVA recombinant viruses expressing HIV-1 SOSIP proteins, the corresponding *AMC011 SOSIP.v5.2* (from clade B) and *ZM197 SOSIP.v5.2* (from clade C) genes were first cloned from original pPPI4 plasmids (14, 15) into the VACV insertional vector pCyA20 under the control of the synthetic early/late virus promoter to construct the pCyA20-AMC011 and pCyA20-ZM197 plasmid transfer vectors, respectively. Then, the corresponding MVA-AMC011 and MVA-ZM197 recombinant vectors were generated following a protocol similar to the one used for the generation of MVA-GP120C14K recombinant vector described above. Purity of MVA-AMC011 and MVA-ZM197 was confirmed by PCR and DNA sequencing.

### Time-Course Expression of GP140 Proteins in MVA-AMC011 and MVA-ZM197 Infected Cells

DF-1 cells were infected with MVA-AMC011, MVA-ZM197, and MVA-wt viruses at 5 PFU/cell and harvested at different times (0, 4, 8, and 24 hpi). Infected cells were collected, and the cells extracts were fractionated by 10% SDS-PAGE and then analyzed by Western-blot using rabbit anti-GP120 antibody (1:3,000; CNB) as described above.

### Fractionation of Purified MVA Virus

Sequential disruption of viral membrane and core-associated proteins of MVA-GP120C14K and MVA-GP120C was carried out using various detergent treatments as previously described (27). After separating an aliquot from sucrose-purified virus stock, referred as total extract (TE), virions were resuspended through sonication in 0.2 ml of Tris-buffer (50 mM Tris-HCl pH 8.5, 10 mM MgCl_2_) containing the non-ionic detergent NP-40 (1%). All the treatments were carried out at 37°C for 30 min. The E1 fraction (soluble lipid envelopes) was removed by centrifugation, while the pellet was re-suspended in 0.2 ml of Tris-buffer −1% NP40 with 50 mM DTT. The E2 fraction (soluble protein matrix-like membranes) was separated by centrifugation and the pellet was re-suspended in 0.2 ml of the previous buffer with the addition of 0.5% sodium deoxycholate and 0.1% SDS. The E3 fraction (soluble core proteins) was removed by centrifugation and the pellet containing the remaining core (Core) was re-suspended in 0.2 ml of lysis SDS-buffer. All the fractions collected were run on SDS-PAGE under reducing conditions and the HIV-1 envelope protein was identified by Western blot.

### Reactivity of GP120C14K Fusion Protein Expressed From Recombinant MVA to bNAbs by Flow Cytometer

The reactivity of GP120C14K fusion protein expressed by MVA-GP120C14K and SOSIP proteins from their corresponding MVA recombinants to human bNAbs was assessed by flow cytometry using a panel of human bNAbs targeting quaternary V1/V2 N-glycans (PGT145, PG16, and PG9), V3 N-glycans (10-1074, PGT121, and 257-DIV), CD4 binding site (VRC01, VRC03, and b12), GP120/GP41 binding region (PGT151, 3BC176, and 35O22), N-glycans (2G12) and membrane-proximal external region (MPER) (10E8) epitopes on the native Env protein. bNAbs were obtained through the AIDS Reagent Program, Division of AIDS, NIAID. HeLa cells were infected with MVA-GP120C14K, MVA-AMC011 SOSIP, MVA-ZM197 SOSIP, or MVA-gp145-GPN [recently described in (28)] recombinant viruses at 3 PFU/cell. At 16 h.p.i., cells were rinsed with PBS (no calcium/magnesium), dissociated with 2 mM EDTA in PBS 1X, washed with FACS buffer (1% BSA, 2 mM EDTA in PBS 1X) and pelleted at 1,500 rpm for 5 min. Cells were then stained with live/death fixable red dye (1:200; Invitrogen) for 30 min at 4°C in the dark, washed twice with FACS buffer and blocked with 3% BSA for 30 min at 4°C. Then, 10 μg/ml in 50 μl FACS buffer of each primary human IgG anti-Env bNAb was used to stain 10^6^ cells for 30 min at 4°C in the dark. Cells were then washed twice with FACS buffer and secondary F(ab′)2-goat anti-human IgG (H+L)-PE antibody (1:200; Beckman Coulter) in 50 μl FACS buffer was added onto the cells. After 30 min incubation at 4°C in the dark, cells were washed twice with FACS buffer and fixed with 0.5% formaldehyde. Samples were acquired in a FC500 1 Laser flow cytometer (Beckman Coulter) and data analyses were performed using FlowJo software (Version 10.4.2; Tree Star, Ashland, OR). Geometric Mean Fluorescence Intensity (gMFI) values on the “live cells” gate were used to analyze the results.

### Animal Immunizations

For mouse immunizations, the animal procedures were granted prior approval by the Ethical Committee of Animal Experimentation of Centro Nacional de Biotecnología (CEEA-CNB), in accordance with 2010/63/UE International EU Guidelines on protection of animals used for experimentation and other scientific purposes, Spanish National Royal Decree RD 53/2013 and Spanish National Law 32/2007 on animal welfare, exploitation, transport, and sacrifice (permit number PROEX 014/15).

All mouse experiments were carried out in groups of female BALB/c mice (H-2^d^), 6–8 weeks old, obtained from ENVIGO Laboratories. The different immunizations carried out to assay the immunogenicity of the GP120C14K fusion construct compared with its controls involved the injection of 2 × 10^7^ PFU of recombinant or wild-type MVA virus per mouse by intra-muscular (i.m) route and of 20 μg of adjuvanted envelope protein per mouse by intra-dermal (i.d) route in groups of four animals each. HIV-1 envelope proteins were adjuvanted in a final inoculum containing 10 μg ODN 1826 (Invivogen), 2% Alhydrogel (Invivogen) and equal parts of Sigma adjuvant Oil (Sigma-Aldrich). At the end of each immunization study, animals were sacrificed using CO_2_ and blood, spleen and popliteal, inguinal, iliac, and sacral draining lymph nodes (DLNs) of interest were extracted from the animals. Comparisons between the MVA viruses MVA-GP120C14K, MVA-AMC011 and MVA-ZM197 for their ability to induce antibodies against the HIV-1 envelope was assayed *in vivo* by immunizing 1 × 10^7^ PFU i.m of each virus in two doses (prime at week 0 and boost at week 4) in groups of six mice each. Blood was extracted 10 days post-boost for antibody measurements in the serum.

Immunogenicity studies in rabbits were performed by ProteoGenix (France). Groups (*n* = 4) of white New Zealand rabbits were immunized using prime/boost protocols with a combination of two doses of MVA recombinants followed by two doses of envelope proteins. At weeks 0 and 4, animals received an i.m. inoculation of 1 × 10^8^ PFU/rabbit of recombinant MVA viruses expressing either GP120C14K (clade C), GP140AMC011 SOSIP (clade B), or GP140ZM197 SOSIP (clade C) proteins. At weeks 8 and 12, animals received an i.m. booster of 20 μg of purified protein (GP120C14K, AMC011, and ZM197) mixed with 10 μg of MPLA adjuvant, corresponding to the same proteins expressed by each of the recombinant MVA vectors. Two weeks after each time point (weeks 2, 6, 10, and 14), blood samples were collected and their serum was extracted for the analysis of antibodies against HIV-1 Env and for neutralization analysis of pseudoviruses. All animals were sacrificed at week 14.

### Analysis of Env-Specific Immune Responses by Intracellular Cytokine Staining (ICS) Assay

#### Analysis of CD8 T Cell Immune Response

Env-specific CD8 T cell responses were analyzed by ICS assay and flow cytometry using previously described protocols (7, 29). Briefly, 4 × 10^6^ splenocytes or 10^6^ cells from DLNs were stimulated with 5 μg/ml of clade C HIV-1 Env-1 peptide (a H-2^d^-restricted CTL epitope with the sequence PADPNPQEM as previously described (30) along with 1 μl/ml GolgiPlug (BD Biosciences), anti-CD107a-Alexa 488 (BD Biosciences) and monensin (1X; eBioscience), in RPMI 1640-10% FCS at 37°C for 6 h in a 96-well plate. Next, the cells were washed, stained for the surface markers, fixed, permeabilized (Cytofix/Cytoperm kit; BD Biosciences) and mixed with anti-CD16/CD32 (BD Biosciences) for blocking the Fc receptors. Cells were stained intracellularly with antibodies against intracellular cytokines conjugated with the appropriate fluorochromes. Live cells were selected using the violet LIVE/DEAD stain kit (Invitrogen). Cells were stained with the following conjugated antibodies: CD3-PE-CF594, CD4-APC-Cy7, CD8-V500, IFN-γ-PE-Cy7, IL-2-APC, and TNF-α-PE and then acquired through a GALLIOS flow cytometer (Beckman Coulter). Data analysis was carried out with FlowJo (Version 10.4.2; Tree Star, Ashland, OR) and the response to specific stimuli was obtained after corrections made with values obtained in unstimulated control samples.

#### Analysis of Germinal Center (GC) B Cells

10^6^ cells from the DLNs were centrifuged and live cells were marked using FVS 520 (BD Biosciences). Next, the cells were washed and mixed with anti-CD16/CD32 (BD Biosciences) for blocking the Fc receptors, and then incubated with 0.3 μg of biotinylated GP120C protein (prepared using the biotin-XX microscale protein labeling Kit; Thermo Fisher) for 30 min at 4°C. After washing, the different markers used for the gating strategy (CD19^+^, B220^+^, IgD^−^, CD95^+^, CD38^−^, IgG1^+^, GL7^+^) were stained using the following conjugated antibodies: CD19-FITC, B220-PE-Cy7, IgD-APCH7, CD95-PE, CD38-PerCPCy5.5, IgG1-BV421, GL7-Alexa647, and Av-PE-CF594, and the cells were acquired through a GALLIOS flow cytometer (Beckman Coulter). Data analysis was carried out using FlowJo software (Version 10.4.2; Tree Star, Ashland, OR).

#### Analysis of T Follicular Helper (Tfh) Cells

For the analysis of the Env-specific Tfh cells, 4 × 10^6^ splenocytes were stimulated with 10 μg/ml of Env-1 peptide and 0.5 μg/ml of GP120C protein. After 18–20 h of incubation, live cells were marked using FVS 520 (BD Biosciences), and then the markers corresponding to the gating strategy (CD3^+^, CD4^+^, CD8^−^, PD1^+^, CXCR5^+^) were stained with the following conjugated antibodies: CD3-PerCPCy5.5, CD4-Alexa700, CD8-eFluor450, PD1-APCeFluor780, CXCR5-PE-CF594, CD134 (OX40)-PE, and CD25-PECy7. Antigen specific Tfh were defined by the upregulation of OX40 and CD25. Cells were then acquired through a GALLIOS flow cytometer (Beckman Coulter). Data analysis was carried out using FlowJo software (Version 10.4.2; Tree Star, Ashland, OR).

### HIV-1 Neutralizing Antibodies in Rabbits Immunized in MVA Prime/Protein Boost Protocols

Neutralization potency was determined as the median antibody concentration required to inhibit HIV pseudovirus activity by 50%. These inhibitory concentration 50% titers (IC50) of neutralizing antibodies against HIV-1 in serum samples from immunized rabbits (corresponding to inverse dilution of sera inducing 50% decrease virus replication) were performed by the TZM-bl assay using a range of pseudotyped viruses carrying envelopes of HIV-1 from clades B and C. Three different laboratories were involved in the neutralization studies. Moog's lab included the following panel of heterologous tier 1 and tier 2 viruses: tier 1, MW965.26 (clade C) and SF162 (clade B); tier 2, BG505.W6M.ENV.C2 (clade A) and sC22 (clade C). Pre-immune serum and inhibition with MLV pseudovirus were used to determine the contribution of non-specific background. Sanders's lab included the following tier 2 viruses: AMC011 (clade B), ZM197 (clade C), REJO4541.67 (clade B), BG505.T332N (clade A), and MLV as negative control. Montefiori's lab included a global panel of tier 2 pseudoviruses.

### Statistical Methods

For the analysis of ICS data, a statistical approach that adjusts the values for the non-stimulated controls (RPMI) and calculates the confidence intervals and *p*-values was used (29). Only antigen responses significantly higher than the corresponding RPMI samples are represented. All of the values represented are background-subtracted. For the determination of the statistical relevance in the ELISA analysis of serum samples, Kruskal-Wallis and Wilcoxon tests were performed.

## Results

### The Fusion Protein GP120C14K Forms Hexamer-Like Structures

**Figure 1:**
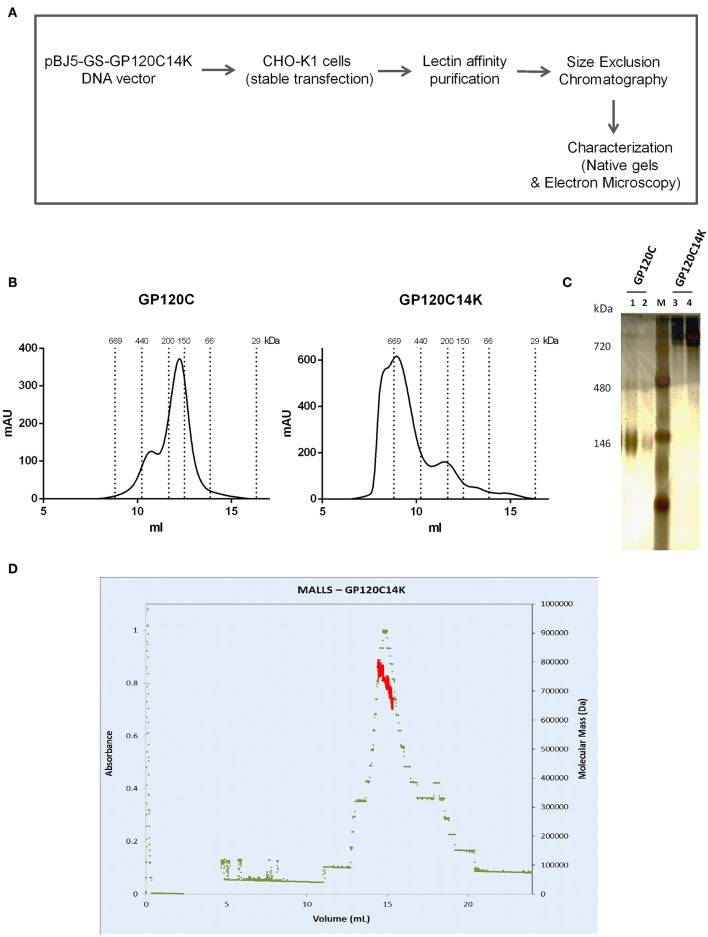
Production and characterization of recombinant proteins. **(A)** Scheme of production and characterization of the GP120C14K protein. **(B)** Size exclusion chromatograms of GP120C (left) and GP120C14K (right) purified proteins. **(C)** Electrophoresis of GP120C (lane 1: 1,000 ng, lane 2: 500 ng) and GP120C14K (lane 3: 500 ng, lane 4: 1,000 ng) proteins under native/non-reducing conditions (4–15% gel, silver stained); Lane M: Native marker. **(D)** Elution profile (elution volume against absorbance) of GP120C14K obtained through Size Exclusion Chromatography Coupled Multi-Angle Light Scattering (SEC-MALLS) technique.

The fusion protein GP120C14K was produced by fusing the HIV-1 *GP120* gene (GP120C; clade C, strain CN54) to the N-terminal truncated VACV *A27L* gene coding for amino acids 28–110 of the viral protein 14K that contribute to the post-translational formation of a trimeric coiled-coil region (5). This fusion gene *GP120C14K* and the *GP120C* genes were sub-cloned into the pBJ5-GS plasmid and subsequently used for transfection of the CHO-K1 cell line and generation of the corresponding stable cell clones. These CHO-K1 cells were grown and GP120C14K and GP120C proteins present in the supernatant, were purified by exploiting the high affinity between lectin (derived from *Galanthus nivalis*) and the mannose sugars present on the glycosylated GP120C protein. Final purification step was carried out using size exclusion chromatography (methodology depicted in Figure 1A). Figure 1B shows the size exclusion chromatography profiles of GP120C (left) and GP120C14K (right) purified proteins.

Following size exclusion, the presence of the purified proteins GP120C and GP120C14K in the selected fractions was analyzed by electrophoresis under native conditions (Figure 1C). The GP120C protein displayed characteristics of a monomeric protein, while the fusion protein GP120C14K was detected at a higher molecular weight (around 700 kDa) suggesting its existence as an oligomer (Figure 1C). The molar mass of GP120C14K obtained through SEC-MALLS technique, suggested a molecular weight of about 700 kDa (Figure 1D). Standard protein stability assays of the GP120C14K protein were performed with 0.2 mg/ml of the protein prepared under different buffer conditions, analyzed for melting temperatures using Thermofluor assay and propensity to form aggregates with Prometheus instrument. Aggregate formation was not detected (see Table S1), suggesting that the protein is largely soluble in the buffers tested. Since the melting temperatures obtained revealed no major transition (ranging between 56 and 58°C), it indicates that the GP120C14K protein is stable.

**Figure 2:**
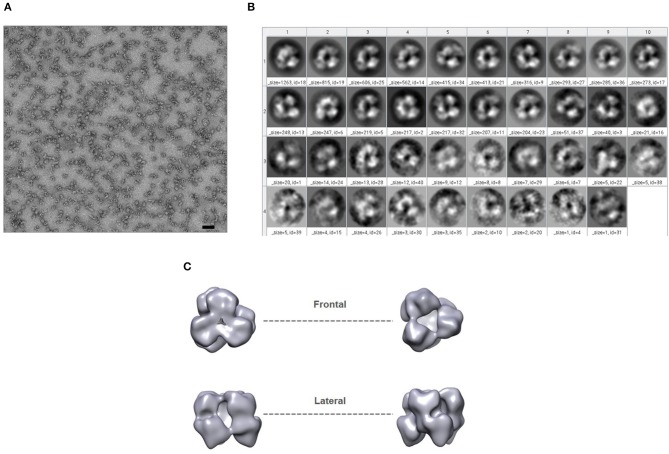
GP120C14K fusion protein forms hexamer-like structures. **(A)** Negative-stain EM analysis of GP120C14K particles (the scale bar corresponds to 50 nm). **(B)** 2-D classification (frontal view) of images. **(C)** 3-D reconstruction of the proposed GP120C14K hexameric structure.

In addition, negative-stain EM analysis was performed to gain structural insights into GP120C14K fusion protein. The protein was prepared using the Gradient Fixation (GraFix) method and images were obtained (Figure 2A). Following 2-D classification of the images obtained (Figure 2B), the three-dimensional representation of the GP120C14K protein was generated (Figure 2C). A closer look at the frontal and lateral views of the model suggests that the protein may exist as a hexamer. The arrangement of the molecule appears to be such that one trimeric unit of the GP120C14K molecule is superimposed on another to form the hexameric structure. This model is in concordance with the relatively high molecular weight of the protein (>700 kDa) observed in the native gels.

### Binding of GP120C14K Fusion Protein to Standard HIV-1 bNAb

**Figure 3:**
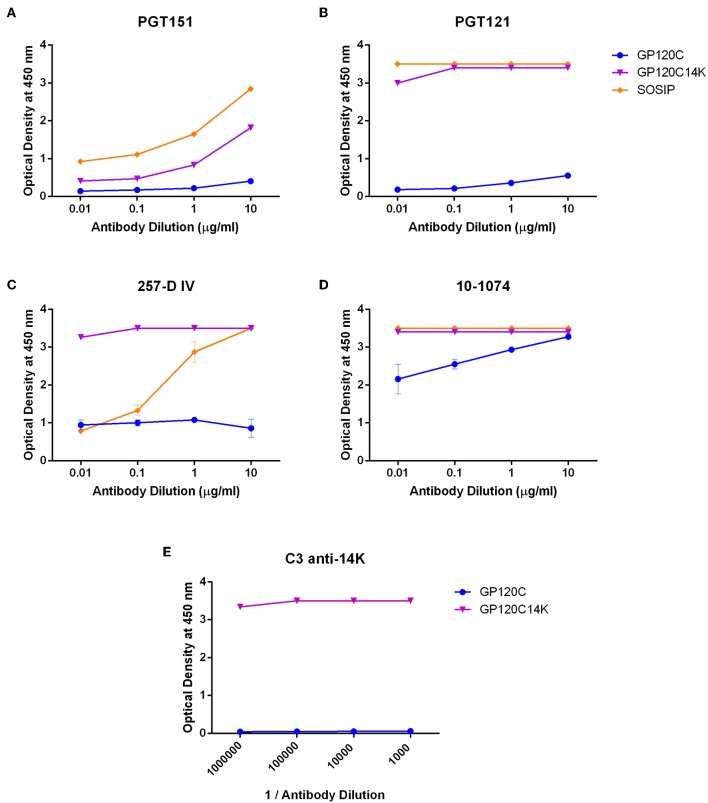
Reactivity of GP120C14K fusion protein to bNAbs. Recognition of GP120C14K, GP120C, and BG505 SOSIP proteins to the quaternary conformational HIV-1 bNAb PGT151 **(A)**, broadly neutralizing antibodies PGT121 **(B)**, 257-D IV **(C)** and 10-1074 **(D)** and to the neutralizing monoclonal antibody C3 directed against the VACV 14K protein **(E)**.

In order to determine the antigenic characteristics of GP120C14K, we next examined the ability of the protein to bind to standard HIV-1 bNAbs. ELISAs to test binding to such antibodies were performed with the purified GP120C14K protein, its monomeric control GP120C and an HIV-1 BG505 SOSIP.v5.2 (15), (referred to as simply SOSIP hereon) native-like trimeric envelope protein as a positive control. The binding characteristics of the different proteins were first determined for the quaternary bNAb PGT151 which is directed to GP120-GP41 interface (31). The highest binding affinity was observed with the purified native-like SOSIP trimeric protein, followed by the GP120C14K protein (Figure 3A), suggesting that the oligomeric GP120C14K protein exposes this key quaternary epitope implied in antibody-based neutralization. The monomeric GP120C showed relatively low binding to PGT151. Next, the binding characteristics of these proteins were tested with PGT121 (32), which is directed to specific glycans at the N332 position of the envelope trimer. In this case, strong binding to PGT121 was detected in both the SOSIP trimer and the GP120C14K protein (Figure 3B), suggesting that both proteins expose the corresponding epitope for this neutralizing antibody. Again, the monomeric GP120C showed relatively low binding to PGT121. Furthermore, the reactivity of the proteins to neutralizing antibodies directed to the V3 variable region of the HIV-1 envelope spike such as 257-D IV (33, 34) and 10-1074 (targeting a carbohydrate dependant epitope on the V3 loop) (35, 36) were determined (Figures 3C,D, respectively). The GP120C14K protein showed the highest reactivity to 257-D IV (Figure 3C) with the similar affinity to 10-1074 as SOSIP protein (Figure 3D), demonstrating the presence and/or exposure of the corresponding epitopes from the V3 region. The monomeric GP120C showed again the lowest binding affinity to both bNAbs.

Similar binding assays were performed against other bNAbs such as PG9 (37) and PG16 (38) (quaternary bNAbs directed to the V2 loop region of the HIV-1 envelope), PGT145 (39) (a quaternary bNAb against the V1–V2 region of the GP120 protein) and VRC01 (40) (directed to the CD4 binding site). Apart from binding to the SOSIP construct, these antibodies showed no significant binding to GP120C14K fusion protein (data not shown). It should be pointed out that due to the ELISA conditions, where the Env protein is fixed to the plate which might in turn affect the structural conformation of the bound protein, reactivity of the fused protein with some of the bNAbs, particularly those recognizing quaternary conformations, might be lost.

As expected, the GP120C14K protein reacted strongly with the VACV 14K specific virus neutralizing monoclonal antibody C3 (41), while the antibody does not show any reactivity for the GP120C protein (Figure 3E).

### Generation and *in vitro* Characterization of MVA-GP120C14K Recombinant Virus

**Figure 4:**
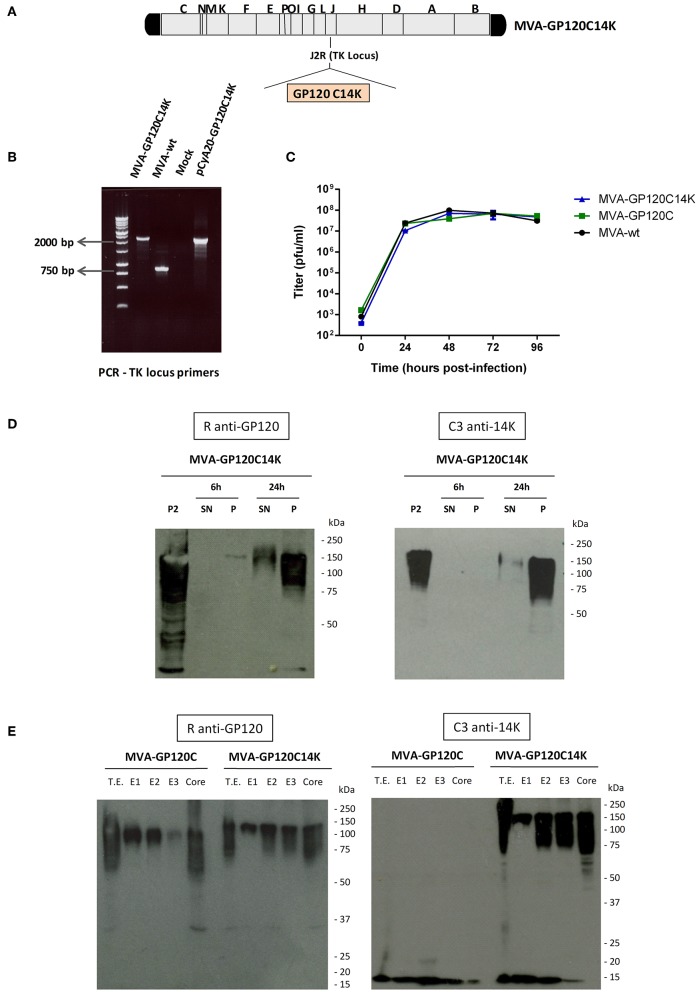
Generation and *in vitro* characterization of MVA-GP120C14K recombinant virus. **(A)** Scheme of the MVA-GP120C14K virus genome showing the insertion of the GP120C14K gene within the TK locus of the parental MVA genome. **(B)** PCR amplification of the viral DNA extracted from DF-1 cells infected with MVA-GP120C14K or MVA-wt viruses using the TK locus primers. The difference in size between the amplified segments from both viruses indicates the correct insertion of GP120C14K gene (2,215 bp) into the TK locus of MVA. The pCyA20-GP120C14K plasmid containing the fusion insert was used as a positive control template for the PCR reaction. **(C)** Growth kinetics of MVA-GP120C14K compared with that of MVA-GP120C and MVA-wt viruses in DF-1 cells (multiplicity of infection 0.01 PFU/cell). **(D)** Time-course expression of GP120C14K antigen by Western blot analysis. DF-1 cells were infected with MVA-GP120C14K virus at 5 PFU/cell and harvested at different times post-infection (6 and 24 hpi). Antigen expression was analyzed as reactivity against HIV-1 GP120 and VACV 14K proteins in the supernatant and cellular pellet (indicated as SN and P, respectively). The P2 stock of the virus was used as a positive control. **(E)** Sub-virion localization of GP120C and GP120C14K proteins by fractionation of the sucrose-purified MVA-GP120C and MVA-GP120C14K virus preparations, respectively. The unfractionated lysate virions (T.E.) and the various fractions (E1, E2, E3, and Core) obtained after sequential disruption by detergents were analyzed by Western-blot. Reactivity against HIV-1 GP120 and VACV 14K (bands corresponding to higher m.w being GP120C14K and lower m.w being 14K) are shown.

In order to develop a viral vector system for the delivery of GP120C14K protein and the analysis of its immunogenic characteristics, an attenuated recombinant MVA virus expressing the fusion protein GP120C14K was constructed (Figure 4A). The correct insertion of the GP120C14K gene into the TK locus of the MVA genome was confirmed by PCR amplification using primers that cover the TK-flanking regions (Figure 4B). The larger fragment obtained corresponds with the expected size of the GP120C14K insert, and no wild-type contamination is observed in the viral preparation. In addition, the GP120C14K insert present in the MVA virus genome was stable as determined after nine consecutive passages from the initial crude preparation—P2 viral stock (data not shown).

The growth kinetics of the virus MVA-GP120C14K was compared with its parental MVA-wt and with the recombinant MVA-GP120C virus. As it is observed in Figure 4C, the growth kinetics of the three viruses analyzed are similar, indicating that the insertion of GP120C or GP120C14K genes does not affect the MVA viral growth.

The antigen expression by MVA-GP120C14K virus was determined by Western blot analysis from DF-1 cells infected with MVA-GP120C14K virus and harvested at different times post-infection (Figure 4D). Expression of GP120C14K was detected with anti-GP120 antibody (Figure 4D, left panel) and with anti-14K antibody (Figure 4D, right panel) in cell lysates and supernatant. The level of GP120C14K protein increased markedly with detectable levels in the supernatant at 24 h post-infection (Figure 4D).

Since VACV 14K protein is localized in the membrane of the mature virus (MV) (27), it was of interest to know whether GP120C14K fusion protein was also localized within the purified virion. Therefore, sucrose-purified MVA-GP120C and MVA-GP120C14K virus preparations were subjected to sequential disruption using different detergent treatments to obtain the various fractions: E1 (NP40, membrane), E2 (DTT, protein matrix-like membrane), E3 (DOC, soluble core proteins), and Core (SDS, insoluble core material). The fractions were analyzed for the presence of GP120C or GP120C14K proteins by Western-blot using antibodies against GP120 (Figure 4E, left panel) or 14K (Figure 4E, right panel). These blots revealed an association of the recombinant GP120C14K fusion protein with the virion and its differential compartmentalization within the virus membrane. This is consistent with previously described studies where the association of antigens expressed from vaccinia virus vectors are distributed differentially according to the nature of the protein (27).

### MVA Infected Cells Express GP120C14K and GP140 SOSIPs That Show Similar Reactivity to bNAbs

**Figure 5:**
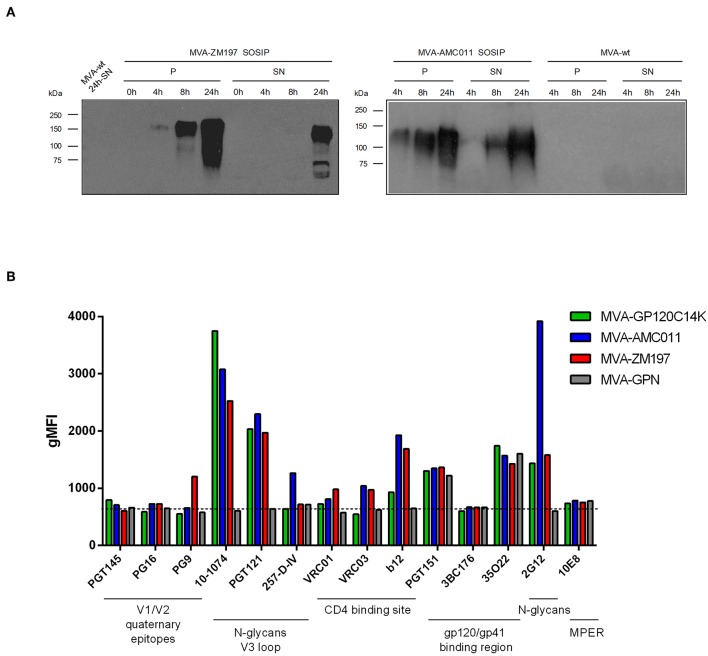
Time-course expression of HIV-1 Env SOSIP proteins expressed from MVA vectors by Western-blot analysis. **(A)** Monolayers of DF-1 cells were infected at 5 PFU/cell with MVA-WT, MVA-ZM197 SOSIP, or MVA-AMC011 SOSIP viruses. At the indicated times post-infection, infected cells were collected, cells extracts fractionated by 10% SDS-PAGE and analyzed by Western-blot using rabbit polyclonal anti-GP120 antibody to evaluate the expression of the SOSIP proteins in cellular pellet (P) and supernatant (SN). **(B)** bNAbs binding profile to Env antigens by flow cytometry. HeLa cells infected with MVA-GP120C14K, MVA-AMC011 SOSIP, MVA-ZM197 SOSIP, or MVA-GPN viruses were processed for flow cytometry as described under Materials and Methods using 10 μg/ml of the indicated primary human IgG anti-Env bNAb. Samples were acquired in a flow cytometer and geometric Mean Fluorescence Intensity (gMFI) values on the “live cells” gate were used to analyse the results.

Next, we examined whether the bNAb reactivity to GP120C14K, when produced as recombinant protein in MVA-GP120C14K infected non-permissive cells was comparable to that observed with MVA-based recombinant viruses that express two different GP140 proteins, the clade B AMC011 SOSIP.v5.2 or the clade C ZM197 SOSIP.v5.2 (14, 15). These vectors, termed MVA-AMC011 and MVA-ZM197, respectively, were generated using similar methods as for MVA-GP120C14K and correctly expressed the GP140 proteins that were released into the medium of MVA-infected DF-1 cells (Figure 5A). Also included for comparison is the MVA virus co-expressing membrane-bound clade C trimeric GP145 envelope and gag-induced virus like particles, MVA-gp145-GPN.

When HeLa cells were infected with the different MVA-based recombinants and were analyzed by flow cytometry with a panel of bNAbs, we observed similar specificities of the bNAbs for MVA-G120C14K as compared to the two other MVA-AMC011 and MVA-ZM197 SOSIP vectors, although some differences in the extent of binding were observed between the viral vectors (Figure 5B). In the case of 10-1074, a strong reactivity of this antibody to the GP120C14K and to the two SOSIPs was observed, with higher reactivity observed by the fused protein. There was also good reactivity of PGT121 and 2G12 (with high reactivity in particular to MVA-AMC011) to Env proteins expressed by the three MVA vectors (Figure 5B). These findings provided evidence that expression of GP120C14K protein during MVA infection follows similar pattern of bNAbs recognition as to GP140 SOSIP forms expressed from MVA-AMC011 or MVA-ZM197 in infected cells.

Since SOSIPs are GP140 proteins without a transmembrane domain, it should be taken into account that when using virus-infected cells, binding of bNAbs to SOSIPs could be due to released GP140 that binds back via CD4 or lectin receptors; it could also be due to some membrane retention during infection as part of Env binding with membrane components of the virus (in fact when MVA is purified from chick cells, SOSIPs are present in the membrane fraction of the virion; data not shown). In addition, we do not know if Env produced by MVA-infected cells represent a heterogeneous mixture of protein forms (monomeric, dimers, trimers, degradation products), although according to size these proteins are synthesized mainly as full-length products under reducing conditions (Figure 5A).

### A Heterologous MVA Viral Vector Prime/Recombinant Protein Boost Immunization With GP120C14K Triggers Higher Proportions of Env-Specific CD8 T Cells Than the Monomeric GP120C

**Figure 6:**
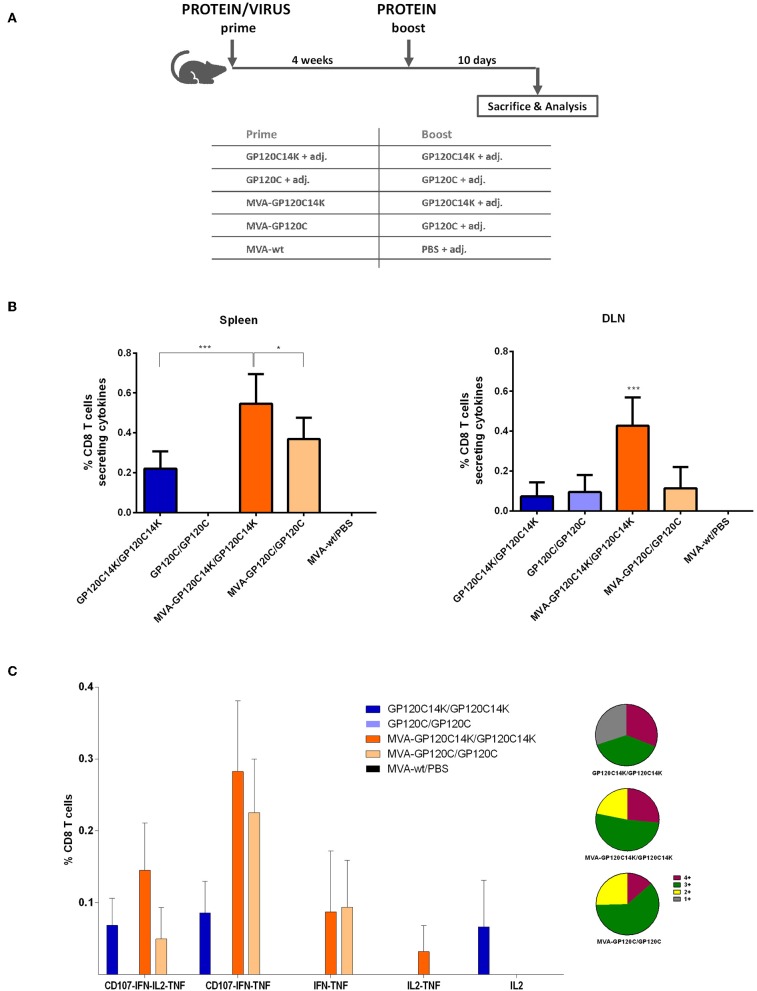
HIV-1 Env-specific CD8 T cell immune responses elicited in spleen and DLNs after M+P or P+P prime/boost immunizations of mice with GP120C or GP120C14K. **(A)** Immunization schedule and immunization groups for the evaluation of the immunogenicity of GP120C14K over GP120C using a protein prime/protein boost (P/P) protocol or an MVA prime/protein boost (M/P) approach. “adj.” refers to the adjuvant mixture used. **(B)** HIV-1 Env-specific CD8 T cell responses measured at 10 days post-boost in the spleen (left) and DLNs (right) represented as the percentage of HIV-1-specific CD8 T cells secreting cytokines and/or expressing CD107a. *p < 0.05, ***p < 0.001. **(C)** Polyfunctional profile of the Env-specific CD8 T cell response at 10 days post-boost in the spleen. Data are represented with their respective confidence intervals for each of the population analyzed. The pie charts represent the relative proportions of cell populations that express four (burgundy), three (dark green), two (yellow), or one (gray) cytokine(s)/CD107a.

To examine the ability of GP120C14K fusion antigen to elicit T cell immune responses against the HIV-1 envelope and compare the same with the monomeric GP120C, *in vivo* studies using BALB/c mice were performed according to the schedule depicted in Figure 6A. A total of four animals per group were immunized (i.d. for the proteins and i.m. for the MVAs) with the corresponding immunogens: protein prime/protein boost (P/P) or MVA virus prime/protein boost (M/P). To determine the HIV-1 Env-specific CD8 T cell responses, splenocytes or cells from DLNs from vaccinated animals were stimulated with an Env-1 peptide. Following stimulation, the percentage of CD8 T cells that secrete one or more of the specific cytokines (IFN-γ, IL-2, and TNF-α) and/or express CD107a (a surrogate marker for cytotoxicity) was analyzed and compared between groups at the peak of response (10 days post-boost).

In general, MVA-prime/protein-boost (M/P) groups showed higher HIV-1 Env-specific CD8 T cell responses than their corresponding protein-only groups (P/P) (Figure 6B). Specifically, the highest magnitude of HIV-1 Env-specific CD8 T cell response was observed with the GP120C14K (M/P) group in both organs analyzed (spleen and DLNs) and the response was statistically significant (Figure 6B).

The quality of the HIV-1-specific CD8 T cell response was measured by the analysis of the polyfunctional profile elicited in splenocytes from the different immunization groups measured by the ability of the cells to secrete more than one type of cytokine and/or to express CD107a. As observed in Figure 6C, groups M/P induced higher polyfunctional responses compared to their corresponding P/P counterparts. High polyfunctional responses were detected in the animals immunized with GP120C14K (M/P) with more than 75% of the HIV-1 Env-specific CD8 T cells exhibiting three or four markers. Env specific-CD8 T cells producing CD107a+IFN-γ+TNF-α and CD107a+IFN-γ+IL-2+TNF-α were the populations most represented by the heterologous M/P vaccination groups.

### Activation of HIV-1 Env-Specific GC B Cells in the DLNs of Immunized Mice

**Figure 7:**
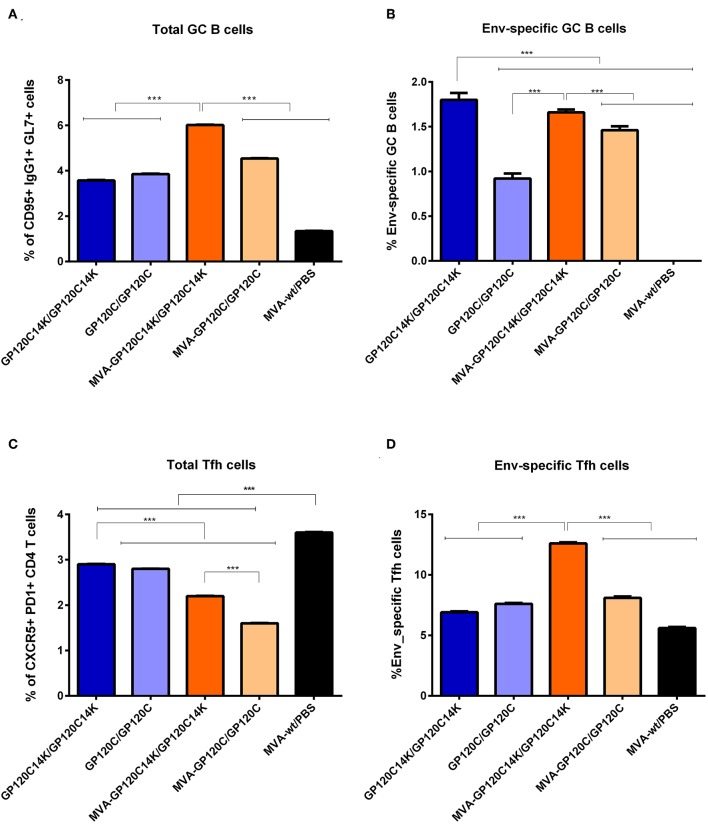
HIV-1 Env-specific GC B cell and Tfh immune responses elicited in DLNs and spleen, respectively, after M/P or P/P prime/boost immunizations of mice with GP120C or GP120C14K. Percentage of total (CD95+ IgG1+ GL7+) **(A)** or Env-specific **(B)** GC B cells in the DLNs of immunized animals. ***p < 0.001. Percentage of total (CXCR5+ PD1+) **(C)** or Env-specific **(D)** Tfh cells in the spleen of immunized animals. ***p < 0.001.

Germinal centers (GCs) are secondary lymphoid structures within B cell follicles where B cells go through affinity maturation and class-switch recombination to generate high-affinity antibodies (42–45). To evaluate the ability of the MVA-GP120C14K/protein GP120C14K immunization regimen to exploit the germinal center reactions for the development of humoral responses to Env, the percentage of Env-specific GC B cells elicited in the DLNs following vaccination was determined by flow cytometry. At 10 days post-boost, the highest percentage of total GC B cells was observed in mice immunized with the GP120C14K following a M/P regimen (Figure 7A). This response was significantly higher (*p* < 0.001) to that obtained with all the other groups (Figure 7A). Within the respective populations of GC B cells, the highest percentage of HIV-1 Env-specific GC B cells was observed with the GP120C14K P/P group, compared to that obtained with the other vaccination groups (*p* < 0.001) (Figure 7B). In general, immunization with the fusion antigen GP120C14K in both protocols P/P and M/P showed significantly higher percentages of Env-specific GC B cells (*p* < 0.001) than the corresponding groups immunized with GP120C.

### Activation of HIV-1 Env-Specific Tfh Cells in the Spleen of Immunized Animals

CD4 Tfh cells are essential for the development and maintenance of GC reactions, a critical process that promotes the generation of long-lived high affinity humoral immunity (46, 47). Since Tfh cells are thought to play an important role in the control of HIV-1, we next defined the degree of activation of this type of immune cells following prime/boost immunization with the GP120C14K antigen. At 10 days post-boost, the percentage of total Tfh cells in the *in vitro* antigen stimulated splenocytes from mice immunized with the protein only GP120C14K group (P/P) was higher than that observed in the M/P group (*p* < 0.001; Figure 7C). Furthermore, the percentages of total Tfh cells induced by the fusion antigen GP120C14K in either protocol (P/P and M/P) were significantly higher (*p* < 0.001) than those observed in the groups immunized with their monomeric GP120C counterparts (Figure 7C). Within the Tfh population, the highest proportion of HIV-1 Env-specific Tfh cells (defined by the expression of CD25 and OX40) was observed in the splenocytes from mice immunized with the GP120C14K fusion antigen under the M/P protocol (*p* < 0.001; Figure 7D).

### HIV-1 Env-Specific Antibodies Induced in Mice by the Different Prime/Boost Immunization Protocols

**Figure 8:**
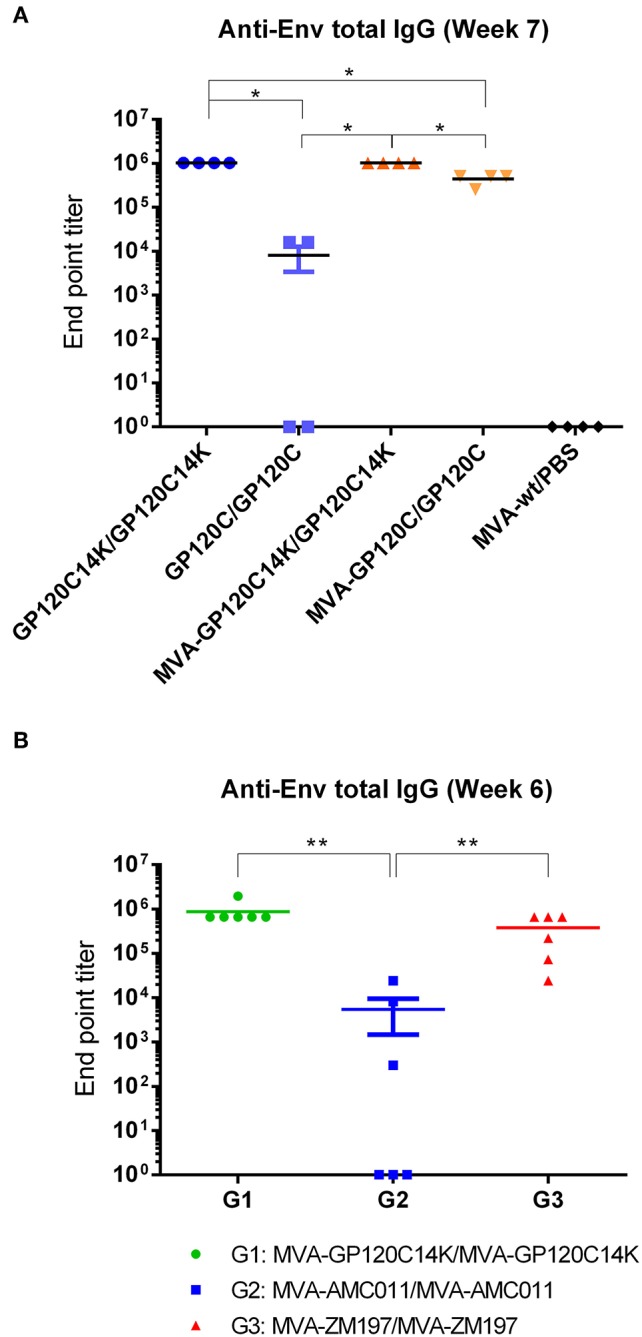
Anti-Env antibody levels from mice immunized with different prime/boost immunization protocols. **(A)** Total IgG in the serum of immunized mice at week 7 measured by ELISA. End point titers of antibody responses against GP120C for each group (*n* = 4) were calculated as the last dilution that provided an optical density value (at 450 nm) that is more than three times the value obtained at the same dilution by the naïve serum. **(B)** Anti-Env antibody levels (total IgG) in the serum of immunized mice (*n* = 6) with different MVA vectors at week 6 measured by ELISA, with end point titers referring to antibody reactivity against the corresponding Env protein from each group. The black bar shown in the plot represents the mean with the SEM of the antibody titers from all the animals from each group. For data in **(A,B)**, pairwise Wilcoxon rank sum test with a Benjamini and Hochberg correction was performed to account for multiple comparisons indicated in the figure, **p < 0.01, *p < 0.05.

The capacity of the immunization protocol of MVA prime and protein boost to raise antibodies (total IgG) against the HIV-1 envelope protein GP120C was analyzed in mouse serum by ELISA at week 7. The end-point titers obtained for the different groups of immunization are shown in Figure 8A. High titers of antibodies against GP120C (1,024,000) were detected in both groups immunized with the fusion antigen GP120C14K (P/P and M/P). While the protein only group with the GP120C monomer (P/P) showed a very low response (100-fold lower) in comparison with that elicited by the fusion protein only group, the GP120C (M/P) group exhibited improved titers of anti-GP120C antibodies (320,000) on an average. In a separate mouse study, the antibody titers against the envelope induced by a MVA-GP120C14K/MVA-GP120C14K protocol were similar to those induced by a homologous MVA-ZM197/MVA-ZM197 immunization protocol, and higher than those induced by the MVA-AMC011/MVA-AMC011 protocol, expressing ZM197 and AMC011 SOSIP proteins (Figure 8B).

### GP120C14K Fusion Protein Induced NAbs Against HIV-1 in Rabbits

**Figure 9:**
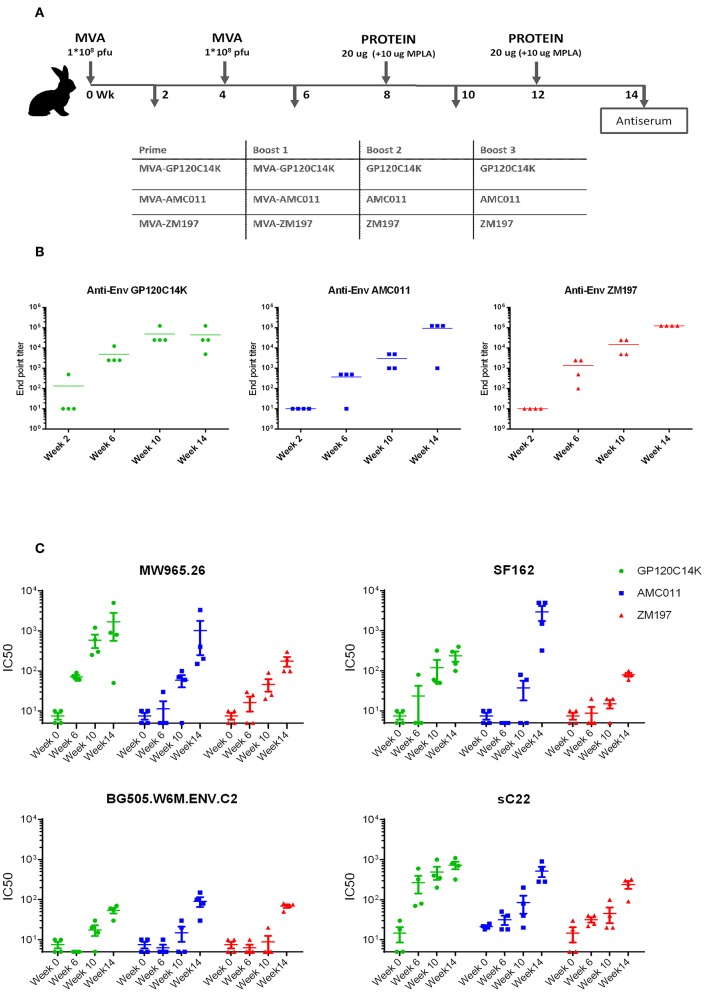
Anti-Env total IgG antibody levels and HIV-1 neutralization capacity in immunized rabbits. **(A)** Immunization schedule and immunization groups (*n* = 4) for the evaluation of the binding antibodies and the neutralization capacity of GP120C14K protein compared with the SOSIP constructs AMC011 and ZM197. **(B)** Anti-Env antibody levels (total IgG) in the serum of immunized rabbits at weeks 2, 6, 10, and 14 measured by ELISA. End-point titers of antibody responses obtained for each group were calculated from the reactivity shown against the corresponding protein and referred as the last dilution that provided an optical density value (at 450 nm) that is more than three times the value obtained at the same dilution by the naïve serum. **(C)** Neutralization titers (IC50) for the different immunization groups at weeks 0, 6, 10, and 14 against HIV-1 tier 1 (MW965.26 and SF162) and tier 2 (BG505 and sC22) viruses.

Induction of neutralizing antibodies against Env protein is a surrogate marker for the control of HIV-1 infection. Thus, we determined in rabbits, the capacity of the prime/boost MVA-GP120C14K/GP120C14K regimen to induce Env specific antibodies with neutralizing capacity against HIV-1. Therefore, rabbits (*n* = 4) were immunized i.m with two doses of recombinant MVA expressing GP120C14K at weeks 0 and 4. For comparison, we used two other MVA recombinants that express GP140 SOSIP proteins, MVA-AMC011 or MVA-ZM197, administered at the same time-points. At weeks 8 and 12, all groups received i.m booster doses with the corresponding purified proteins, either GP120C14K, GP140-AMC011, or GP140-ZM197, adjuvanted with MPLA (Figure 9A). Serum samples were collected at weeks 2, 6, 10, and 14, and analyzed for total anti-Env IgG binding antibodies by ELISA (Figure 9B) and for neutralizing capacity against HIV-1 tier 1 and 2 virus pseudotypes carrying Env by the TZM-bl assay (Figure 9C).

Comparison of total IgG antibody titers revealed high values in all cases with a trend to higher levels in animals immunized with the GP120C14K fusion antigen vs. the SOSIPs (Figure 9B). While the two doses of the corresponding recombinant MVAs elicited moderate levels of anti-Env antibodies, (data from weeks 2 and 6), these responses were markedly boosted with the two doses of the corresponding protein components (data from weeks 10 and 14).

In terms of the neutralizing antibodies, serum from the MVA-GP120C14K/GP120C14K group neutralized (IC50) quite effectively the isolate MW965.26 (tier 1, clade C); even two doses of MVA vector (data from week 6) have neutralizing capacity that is enhanced in a dose-dependent manner by booster doses with the GP120C14K protein (data from weeks 10 and 14). These neutralization values were similar to those triggered by the MVA-AMC011/AMC011 group and superior to those induced by the MVA-ZM197/ZM197 group. In the case of neutralization of SF162 (tier 1, clade B), booster with GP120C14K antigen elicited higher titers than ZM197 but lower than AMC011 (Figure 9C). For the virus isolate BG505 (tier 2, clade A), the neutralization values were similarly induced by the three regimens although with less potency than for MW965.26 and SF162 isolates (Figure 9C). However, the SOSIP AMC011 appears superior to ZM197 and GP120C14K in the neutralization of tier 2 BG505.T332N (see Table S2). Finally, both GP120C14K and AMC011 immunogens induced similar levels of neutralizing antibodies against tier 2 virus (sC22, clade C; Figure 9C). There was no neutralization against a global panel of difficult to neutralize tier 2 viruses in serum of the immunized rabbits for any of the proteins analyzed (data not shown).

## Discussion

As determined in this work and in the previous study (7), fusion of the VACV 14K protein to the HIV-1 GP120 antigen results in the oligomerization of the molecule. The GP120C14K fusion protein described here exists predominantly as a hexamer-like structure, with two trimers positioned together as suggested by EM data. This is consistent with the propensity of trimers of 14K protein to form hexamers, as observed from structure analysis of 14K protein (48). The data from biophysical characterization also indicates the relative absence of protein aggregates in different buffers, suggesting that the oligomeric form is stable and soluble. Oligomerization of the GP120C by the modified 14K protein can provide a favorable immunological quality considering the possibility of more epitopes being presented to the immune system per molecule when used as a vaccine, and the demonstrated adjuvant-like effect conferred by the 14K component in malaria and HIV models (5, 7). The favorable reactivity of the GP120C14K protein to the quaternary antibody PGT151 and to the bNAbs PGT121, 257-DIV, and 10-1074 highlights the presence and/or greater exposure of the corresponding epitopes. While suggesting the antigenic superiority of this protein when compared with the monomer, these results also hint at the possible conformational changes of the GP120 protein induced by the 14K fusion. The comparable reactivity to bNAbs observed in the MVA-GP120C14K infected cells through flow cytometry suggests that the conformation of the GP120C14K fusion protein is relatively similar when expressed from the MVA vector.

Included among the various strategies toward improving the immunogenicity against HIV-1 of MVA-based vectors (49), there is the option to optimize the antigenic form expressed by the poxvirus vector. We decided to follow this strategy and generated the recombinant vector MVA-GP120C14K, which expresses the fusion antigen GP120C14K thus allowing us to evaluate *in vivo* the contribution of the same antigen to the immune responses against the HIV-1 envelope when delivered by both an MVA vector and as a purified protein component. We showed that expression of the GP120C14K fusion antigen by MVA did not alter the replication capacity of the recombinant virus, an important consideration for the large-scale manufacture of viral stocks. Furthermore, we demonstrated that the GP120C14K protein expressed during MVA-GP12014K infection shows a bNAb recognition pattern similar to that observed with GP140 SOSIP forms expressed from MVA-AMC011- or MVA-ZM197-infected cells.

There are evidences indicating that the virus-specific CTLs (CD8^+^ T lymphocytes) help to control HIV-1 replication: the correlation between the decline in viremia and the appearance of HIV-1 specific CD8 T cells (50, 51), the *ex vivo* elimination of HIV-1 infected CD4 T cells by CD8^+^ T cells isolated from HIV-1 controllers (52), or the CD8^+^ T cell-mediated viremia control in simian immunodeficiency virus-infected rhesus macaques (53, 54).

In our mice studies, the group vaccinated with the fusion antigen GP120C14K delivered through MVA prime and boosted with adjuvanted protein exhibited the highest HIV-1 Env-specific CD8 T cell responses, highlighting the capacity of our fusion protein with this prime/boost regimen to mount specific T cell responses against the HIV-1 envelope protein. In addition to the magnitude, higher functionality has been reported to be a key characteristic of CD8^+^ T cells among HIV-1 controllers (55), highlighting the importance of eliciting polyfunctional T cell responses by vaccine candidates. The group vaccinated with the MVA-GP120C14K delivered as a prime and boosted with adjuvanted GP120C14K protein elicited polyfunctional HIV-1 Env-specific CD8^+^ T cell responses that could play a role in anti-HIV-1 Env immunity when provided as vaccination.

It is well-known that Tfh cells are indispensable to the survival, differentiation and proliferation of antigen-specific B cells (46). While it is clear that Tfh cells play a crucial role in the development of neutralizing antibodies, germinal centers and memory B cells (56), the importance of Tfh in the resistance to HIV-1 infection is also under considerable scrutiny. Analysis of HIV-1- infected patients who go on to develop bNAb responses against HIV-1 revealed higher percentages of circulating memory Tfh cells (PD-1^+^ CXCR3^−^ CXCR5^+^), hinting a potential role of Tfh cells in the development of potent neutralizing responses against the virus (57). Furthermore, recent evidence from HIV-1 controllers have shown a positive association between the presence of the resident anti-HIV-1 memory B cells (that possibly contribute to the long-term viremia control) and the HIV-1 Env-specific circulating Tfh cells, highlighting the critical role played by Tfh in helping HIV-1 control and the benefit for inducing such responses through vaccination (58). In our analysis, mice immunized with the fusion antigen GP120C14K through MVA prime and protein boost showed the highest percentage of Env-specific Tfh cells, as defined by the expression of CD25 and OX40, a previously described correlate for antigen-specific Tfh responses (59). This high percentage of Env-specific Tfh cells induced with the fusion antigen hints at the possible role that these cells might be playing in promoting favorable GC reactions toward qualitative humoral responses against HIV-1. These results might indicate the capacity of the fusion antigen, given its specific physio-chemical qualities, to stimulate a response profile that includes, among other unknown cell populations and mechanisms, specific Tfh responses. Since the GC B cells are known receivers of Tfh help, our findings hint at a possible collaboration between these two cellular types that acts favorably toward raising humoral responses against the HIV-1 envelope.

In the case of HIV-1, it has been reported that the quantity and quality of Env-specific Tfh cells in rhesus monkeys with SHIV_AD8_ infection were important in the development of Env-specific IgG-positive GC B cells, which translated to higher cross-neutralizing humoral responses (60). Analysis of the GC B cell responses in the DLNs in our study revealed a high percentage of Env-specific GC B cells in the group immunized with the GP120C14K fusion antigen in the heterologous MVA prime/protein boost protocol (surpassed only by the homologous fusion protein only group). Taken together with the results of the Env-specific Tfh cells, this group shows the best indicator of a favorable HIV-1 Env-specific GC reaction. Further analysis of B cell through ELISPOT could also be a good indicator of the potential improvement to envelope-specific B cells with the use of the GP120C14K fusion antigen.

One of the principal forms of protection against HIV-1 derives from the ability of vaccines to drive the development of potent anti-HIV-1 antibody responses. In our study, mice that received the heterologous immunization with the fusion antigen regimen MVA-GP120C14K/GP120C14K induced high titers of binding antibodies -in the order of 10^6^- against the GP120 protein of the HIV-1 clade C. Recently, such high titers of anti-HIV-1 binding antibodies have been reported in rabbits immunized with native-like envelope trimer molecules (BG505 SOSIP) in various prime-boost combinations (61). The contribution of the fusion protein toward the development of high titers of antibodies against GP120 can be appreciated in mice from the fact that the adjuvanted protein on its own (prime and boost) is also able to induce high titers (in the order of 10^6^) of anti-GP120 binding antibodies, a magnitude that is 10^2^ fold higher compared to the titers obtained with the adjuvanted GP120C protein alone, and that is equivalent to those obtained when the MVA-GP120C14K was administered as the prime component. A similar trend of higher titers of antibodies against the envelope was observed in serum of rabbits immunized with two doses of MVA-GP120C14K, when compared to immunization with MVA vectors expressing SOSIP proteins AMC011 and ZM197, as well as after a single booster dose with purified proteins (Figure 9B). Hence, in terms of antibody stimulation, GP120C14K appears to be a more potent immunogen than the AMC011 and ZM197 SOSIP proteins. This is likely due to the formation of hexamer-like structures of GP120C14K vs. SOSIP trimers.

One of the most important determinations of the immunogenic capacity of an antigen lies in the quality of the antibody response generated, evaluated in terms of their ability to neutralize the viral strain. Regarding this, Sanders et al. have previously reported the generation of autologous tier 2 neutralizing antibody responses in rabbits immunized with native-like SOSIP envelope trimers (62). A comparison of the HIV-1 neutralizing antibodies induced in rabbits immunized with the combination of MVA-GP120C14K/GP120C14K vs. animals immunized with two other recombinant MVA vectors expressing SOSIPs (MVA-AMC011/AMC011 and MVA-ZM197/ZM197) SOSIP proteins revealed, in all cases, the production of NAbs against some HIV-1 tier 1 and tier 2 pseudoviruses. The group immunized with MVA-GP120C14K/GP120C14K (clade C) induced antibodies that neutralized homologous viruses MW965.26 (clade C, tier 1) and sC22 (clade C, tier 2), and the heterologous viruses SF162 (clade B, tier 1) and BG505.W6M.ENV.C2 (clade A, tier 2). The groups immunized with MVA-AMC011/AMC011 (clade B) and MVA-ZM197/ZM197 (clade C) induced neutralization against their corresponding homologous viruses (SF162 in the case of AMC011; MW965.26; and sC22 in the case of ZM197) as well as the heterologous virus BG505. W6M.ENV.C2 (clade A), where MVA-AMC011/AMC011 was the most potent group. However, we did not observe neutralization of rabbit serum against a global panel of difficult to neutralize tier 2 viruses (not shown). Furthermore, a more detailed analysis of the neutralizing antibodies detected in immunized rabbits to include their mapping (through pepscan and scaffolding techniques), variations in isotypes and the glycosylation profiles will be necessary to determine their relevance and quality for further such studies.

While with the current MVA vectors expressing HIV-1 Env we cannot control the relative abundance of native Env forms vs. non-native forms during virus infection, the MVA-GP120C14K vector has the added advantage of triggering strong Env-specific T cell immune responses, which together with its NAb activity should help in the control of HIV-1 infection. Future MVA vectors should be generated with the aim to produce highly stable native-like Env trimers and/or hexamers as a predominant product, with the ability to trigger NAb responses against a broad spectrum of heterologous HIV-1 viruses in prime/boost protocols. In addition, immunization protocols can be optimized to include a late booster dose to re-stimulate the antibody and T cell responses with a view to improving the immunogenicity elicited against HIV-1.

In summary, fusion of the VACV 14K protein oligomerizes the HIV-1 GP120C protein forming hexamer-like structures. The immunization regimen delivering the MVA-GP120C14K as a prime and GP120C14K protein as a boost elicits in immunized mice stronger HIV-1 Env-specific immune responses, as quantified by CD8^+^ T cells, Tfh, GC B cells and anti-GP120C antibody titers, when compared to the delivery of the monomeric GP120C. In rabbits, MVA-GP120C14K/GP120C14K prime/boost regimen triggers NAbs against several HIV-1 tier 1 and tier 2 viruses comparable to NAb levels induced by SOSIPs delivered by a similar protocol. Therefore, the immunization regimen MVA-GP120C14K/GP120C14K can be considered as a potential regimen for vaccination against HIV-1. The generation of fusion antigens with vaccinia virus 14K protein can be considered as a strategy for potentiation of immunogenicity against different pathogens.

## Data Availability Statement

The datasets generated for this study are available on request to the corresponding author.

## Ethics Statement

The animal study was reviewed and approved by Ethical Committee of Animal Experimentation of Centro Nacional de Biotecnología (CEEA-CNB).

## Author Contributions

SR, EM-P, CG, JG-A, BP, and ME conceived and designed the project. AV contributed to the initial design of the fusion construct. SR, EM-P, CG, JG-A, BP, and CS-C performed the experiments involving the construction, characterization, and immunological evaluation of the vaccine candidates. CS performed part of the protein purifications. COSS contributed with statistical analysis of the data. MP-R, AC, and JC performed the electron microscopy analysis. JB, AS, and RS contributed to the design, construction and purification of the SOSIP proteins. CM and RS performed neutralization assays with the immunized rabbit sera. SR and ME wrote the manuscript with inputs from several authors. All authors discussed the results, contributed to the interpretation of data and reviewed the manuscript.

### Conflict of Interest

The authors declare that the research was conducted in the absence of any commercial or financial relationships that could be construed as a potential conflict of interest.
