# Supplementary material for: The Envelope-Based Fusion Antigen GP120C14K Forming Hexamer-Like Structures Triggers T Cell and Neutralizing Antibody Responses Against HIV-1
Source: Front Immunol. 2019 Dec 4;10:2793. doi: 10.3389/fimmu.2019.02793 (PMC6904342; doi:10.3389/fimmu.2019.02793)
Supplement: Supplementary file 1 [file Data_Sheet_1.docx]

Table S1. Stability analysis of the GP120C14K protein. The different buffer conditions under which the stability of the protein was analysed through Prometheus (measurement of aggregation events) and Thermofluor assay (measurement of Tm) are indicated. “ND” indicates No Data. All temperatures indicated are in degree Celsius.

| **Condition** | **Description** | **Aggregation** | **Thermofluor Tm** |
| --- | --- | --- | --- |
| 1 | 50 mM MIB, 150 mM NaCl, pH 4.5 | ND | 58 |
| 2 | 50 mM MIB, 150 mM NaCl, pH 5.5 | ND | 58 |
| 3 | 50 mM MIB, 150 mM NaCl, pH 6.5 | ND | 57 |
| 4 | 50 mM MIB, 150 mM NaCl, pH 7.0 | ND | 57 |
| 5 | 50 mM MIB, 150 mM NaCl, pH 7.5 | ND | 57 |
| 6 | 50 mM MIB, 150 mM NaCl, pH 8.0 | ND | 57 |
| 7 | 50 mM MIB, 150 mM NaCl, pH 8.5 | ND | 57 |
| 8 | 50 mM MIB, 150 mM NaCl, pH 9.5 | ND | 57 |
| 9 | 50 mM Hepes pH 7.5, 25 mM NaCl | ND | 57 |
| 10 | 50 mM Hepes pH 7.5, 100 mM NaCl | ND | 57 |
| 11 | 50 mM Hepes pH 7.5, 250 mM NaCl | ND | 57 |
| 12 | 50 mM Hepes pH 7.5, 500 mM NaCl | ND | 57 |
| 13 | 50 mM Tris pH 7.5, 25 mM NaCl | ND | 57 |
| 14 | 50 mM Tris pH 7.5, 100 mM NaCl | ND | 57 |
| 15 | 50 mM Tris pH 7.5, 250 mM NaCl | ND | 57 |
| 16 | 50 mM Tris pH 7.5, 500 mM NaCl | ND | 58 |
| 17 | 100 mM NaPO4 pH 6.5 | ND | 58 |
| 18 | 100 mM NaPO4 pH 7.5 | ND | 57 |
| 19 | 100 mM NaPO4 pH 8.5 | ND | 56 |
| 20 | 50 mM Hepes pH 7.5, 150mM NaCl, 10% glycerol | ND | 57 |
| 21 | 50 mM Hepes pH 7.5, 150mM NaCl, 20% glycerol | ND | 57 |
| 22 | 50 mM Hepes pH 7.5, 150mM NaCl, 0.05% Tween | 78.6 | ND |
| 23 | 50 mM Hepes pH 7.5, 150mM NaCl, 10 mM TCEP | 57.9 | 58 |
| 24 | 50 mM Hepes pH 7.5, 150mM NaCl, 200 mM NDSB201 | ND | 57 |
| 25 | 50 mM Hepes pH 7.5, 150mM NaCl, 5 mM EDTA | ND | 57 |
| 26 | 50 mM Hepes pH 7.5, 150mM NaCl, 20mM MgSO4 | 85.6 | 57 |
| 27 | 50 mM Hepes pH 7.5, 150mM NaCl, 2.5 mM ZnCl2 | ND | 56.5 |
| 28 | 50 mM Hepes pH 7.5, 150mM NaCl, 10 mM MnCl | ND | 56 |
| 29 | 50 mM Hepes pH 7.5, 150mM NaCl, 20 mM LiCl | 57.4 | 56.5 |
| 30 | 50 mM Hepes pH 7.5, 150mM NaCl, 20 mM CaCl | ND | 56.5 |
| 31 | 50 mM Hepes pH 7.5, 150mM NaCl, 200 mM Glu/Arg | ND | 57.5 |
| 32 | 50 mM Hepes pH 7.5, 150mM NaCl, 50 mM NaSCn | ND | 58 |

Table S2. Neutralization (IC50) by TZM-bl assay of different HIV-1 pseudo viruses with serum from rabbits immunized with MVA vectors and protein components. Values are given for each of the HIV-1 pseudo viruses used in the assay, indicating the clade and corresponding tier.

|  |  | **AMC011** | **ZM197M+++** | **REJO4541.67** | **BG505.T332N** | **MLV** |  |  |  |
| --- | --- | --- | --- | --- | --- | --- | --- | --- | --- |
|  | **clade** | B | C | B | A | neg. control |  |  |  |
|  | **tier** | 2 | 1B / 2 | 2 | 2 |  |  |  |  |
| **GP120C14K** | rabbit #1 | <20 | <20 | <20 | <20 | <20 |  |  | 20-40 |
|  | rabbit #2 | <20 | <20 | <20 | <20 | <20 |  |  | 41-100 |
|  | rabbit #3 | <20 | 23 | <20 | 21 | <20 |  |  | 101-1000 |
|  | rabbit #4 | <20 | <20 | <20 | <20 | <20 |  |  | >1001 |
| **AMC011** | rabbit #1 | <20 | <20 | <20 | 171 | <20 |  |  |  |
|  | rabbit #2 | <20 | <20 | <20 | 38 | <20 |  |  |  |
|  | rabbit #3 | 23 | 23 | 238 | 194 | <20 |  |  |  |
|  | rabbit #4 | <20 | <20 | <20 | <20 | <20 |  |  |  |
| **ZM197** | rabbit #1 | <20 | 28 | <20 | 122 | <20 |  |  |  |
|  | rabbit #2 | <20 | <20 | <20 | <20 | <20 |  |  |  |
|  | rabbit #3 | <20 | <20 | <20 | <20 | <20 |  |  |  |
|  | rabbit #4 | <20 | <20 | <20 | 62 | <20 |  |  |  |
